# Welfare Benefits of Intradermal Vaccination of Piglets

**DOI:** 10.3390/ani10101898

**Published:** 2020-10-16

**Authors:** Déborah Temple, Marta Jiménez, Damián Escribano, Gerard Martín-Valls, Ivan Díaz, Xavier Manteca

**Affiliations:** 1 School of Veterinary Science, Autonomous University of Barcelona, 08193 Bellaterra, Barcelona, Spain; gerard.martin@uab.cat (G.M.-V.); xavier.manteca@uab.cat (X.M.); 2 Merck Sharp & Dohme Animal Health, Carbajosa de la Sagrada, 37008 Carbajosa de la Sagrada, Salamanca, Spain; marta.jimenez@merck.com; 3 School of Veterinary Medicine, University of Murcia, 30100 Murcia, Spain; det20165@um.es; 4 Institute of Agrifood Research and Technology–Animal Health Research Center, 08193 Bellaterra, Barcelona, Spain; ivan.diaz@irta.cat

**Keywords:** intradermal vaccination, pig, behaviour, stress, welfare

## Abstract

**Simple Summary:**

Vaccination is reported as a stressful and painful event for animals. Very little is known about the welfare benefits of using needle-free intradermal vaccination in pigs. A commercial trial was conducted in Spain to assess whether intradermal vaccination could improve the welfare of growing pigs. The results demonstrated that the needle-free intradermal injection reduced the behavioural reaction at the time of vaccination. Contrary to intramuscular vaccination, intradermal vaccination did not affect general activity, social behaviour and exploratory behaviour of the piglets after the injection. Blood C-reactive protein and Haptoglobin levels were lower in the intradermal group suggesting a decreased acute phase response and reduced muscular damage compared to intramuscular injection. Thus, the needle-free intradermal method of vaccination of pigs represents a less invasive and less painful alternative to conventional intramuscular injections with needles.

**Abstract:**

Vaccination is reported as a stressful and painful event for animals. This study investigated whether needle-free intradermal vaccination improves the welfare of weaned pigs through the reduction of stress and pain biomarkers and improvement of behavioural parameters compared to traditional intramuscular injection with a needle. A total of 339 weaned piglets were allocated to 3 treatment groups: Intradermal Application of Liquids (IDAL) pigs, vaccinated against Porcine Circovirus type 2 (PCV2) by means of intradermal vaccination using a needle-free device Porcilis^®^ PCV ID; Intramuscular (IM) pigs vaccinated against PCV2 with Porcilis^®^ PCV intramuscularly with a needle; CONTROL pigs were managed identically but did not receive any vaccine injection. At the time of the injection, the reaction of IDAL piglets was similar to control piglets, whereas a greater percentage of piglets vaccinated intramuscularly displayed high-pitch vocalizations (7% CONTROL, 7% IDAL, 32% IM) and retreat attempts (3% CONTROL, 7% IDAL, 39% IM). The day after vaccination, IDAL piglets did not differ from the control piglets for any of the behavioural variables studied through scan samplings. IM piglets showed a lower frequency of social negative interactions (*p* = 0.001) and rope manipulation (*p* = 0.04) compared to the CONTROL group. Resting postures did not differ between treatments. At 28 h post-vaccination, IDAL piglets presented lower blood C-reactive protein levels (CONTROL = 20 μg/mL; IDAL = 39 μg/mL; IM = 83 μg/mL, *p* < 0.0001) and blood Haptoglobin (CONTROL = 1.8 mg/mL; IDAL = 1.9 mg/mL vs. IM = 3.1 mg/mL, *p* < 0.0001) compared to IM piglets. Salivary chromogranin A and alpha-amylase did not differ between treatment groups when measured 25 min post-vaccination. The method of vaccination did not affect the growth of the piglets or their rectal temperature. These results support that needle-free intradermal vaccination reduces vaccination-related pain in growing pigs.

## 1. Introduction

Vaccination is one of the most common and effective strategies to prevent diseases in animals. In commercial pig production, pigs are often vaccinated several times through their life cycle. Intramuscular injection is the most commonly used method of vaccination in pigs.

Pain, necrosis, and self-mutilation have been reported in response to intramuscular injection in animals. Most of those studies have been done in laboratory animals [1]. Welfare-related research on routine husbandry procedures conducted on piglets has focused mainly on castration, tail docking and tooth clipping. Acute pain related to ear tagging and ear notching has been reported by Leslie et al. [2]. However, intramuscular injections of piglets may raise welfare concerns and yet have received limited attention in previous studies.

Beyond classical intramuscular vaccination, alternative routes have been evaluated, including intranasal and oral; however, few vaccines using these routes have been commercialized, most of them against intestinal pathogens, such as *E. coli* or *Salmonella cholerasuis*, by oral administration.

The skin, as a highly effective component of the immune system, is an attractive target for vaccination due to its high density of immunocompetent cells [3]. In humans, needle-free methods by intradermal injection cause less pain and stress at the time of vaccination [4,5]. By analogy, a similar effect should be expected in animals.

Behavioural changes tend to be the most sensitive indicators of an animal’s perception of environmental changes and therefore the degree of stress, pain and discomfort caused by different vaccination methods can be measured through behavioural observations. Behavioural changes have been commonly used to measure pain and distress linked to injections in laboratory animals. For example, the occurrence of site-directed behaviours and/or variations in frequencies of normal behaviours such as foraging, locomotive activity and grooming have been used to measure the degree of pain and distress after injections in laboratory animals [6,7]. Few studies have been done to measure pain-related behavioural responses due to different types of injections in farm animals. Mc Glone 2016 et al. [8] did not observe any difference in pain-related behaviours (lying, eating, sitting, standing, walking and drinking) 1 h post-injection of a saline solution administered either intramuscularly or subcutaneously in pigs. Göller et al. [9] demonstrated that suckling piglets vaccinated intradermally showed more active behaviour and more suckling activity than intramuscularly vaccinated piglets until the day after vaccination. A recent study reported differences in piglets’ vocalizations at the moment of injection between pigs vaccinated intramuscularly and intradermally, where piglets vaccinated intramuscularly showed a greater number of vocalizations with a longer and more powerful peak [10]. Furthermore, sows vaccinated intramuscularly were more fearful two days after vaccination than sows vaccinated with a needle-free system [11].

Vaccination has been shown to cause different degrees of acute phase response, which can be monitored by measuring acute phase proteins (APPs) [12]. APPs have been proposed as suitable biomarkers for monitoring inflammatory response [13]. Although plasma cortisol concentration has been widely used to measure stress in farm animals, recent studies have not found significant differences in cortisol levels after vaccination [10,11] suggesting the importance of investigating alternative stress biomarkers to evaluate the effects of vaccination methods. Salivary alpha-amylase (sAA) and Chromogranin-A (CgA) have been shown to be reliable alternatives to catecholamines for monitoring the sympathetic adrenal-medullary (SAM) activity in pigs, which constitutes the initial and “fast” response to stress and pain [14,15,16,17].

Practical benefits of needle free injections include consistent vaccine delivery as well as greater antigen dispersion and lower vaccine volume. Such systems eliminate the need to dispose used and broken needles and reduce the risk of injuries in people [18]. The presence of broken needles and needle fragments in carcasses is a serious problem for the meat industry [19]. Despite improvements in the detectability of needles in finished products, a significant number of needle fragments are still undetectable in meat [20,21]. Needle-free systems eliminate the risk of residual needles in pig carcasses and meat products. According to several surveys, pig carcasses are damaged to some extent due to improper injections [22,23]. Besides representing a substantial economic loss, the presence of tissue damage at the site of injection in pig carcasses is an indicator that pain occurred. Contrary to Houser et al. [21] who did not report a significant difference in carcass lesions between vaccination methods, carcass defects (such as granuloma, abscess, and fibrosis) resulting from intramuscular injections of vaccines have been reported in some studies [24,25]. Needle-free injections might be expected to prevent possible tissue damage and bacterial contamination caused by needles.

Needle-free devices differ on the power source: spring-powered, battery-powered and compressed-gas-powered [18]. This study will focus on the use of the Intra Dermal Application of Liquids (IDAL, MSD Animal Health, Salamanca, Spain) needle-free injector, which is a battery-powered injector. The present study aimed to evaluate the behavioural and physiological response of weaned piglets to needle-free IDAL vaccination against PCV2 compared to the needle-syringe intramuscular method. Health indicators such as skin reaction at the site of injection, rectal temperature and growth were monitored until 3 weeks post-injection.

## 2. Materials and Methods 

### 2.1. Ethical Statement

The experimental protocol CEEAH 3366 described in this experiment was approved by the Institutional Animal Care and Use Committee of the Universitat Autònoma de Barcelona.

### 2.2. Animal, Housing, and Experimental Procedure 

This study was conducted on a commercial farm in Catalunya. Fifteen pens from a commercial weaner building were used.

A total of 339 weaner pigs were included in the study. Litters from 33 mothers were weaned at approximately 28 days of age and distributed to each pen in groups of 22–24 piglets. Males and females were allocated to different pens and balanced across treatments. Pens (1.5 × 3.9 m, 0.25 m^2^/pig) had plastic, fully-slatted floors and solid plastic. Pigs were fed ad libitum from a weaner hopper with five feeding spaces and water was provided from nipple drinkers. Room temperature was set a 26 °C. An organic rope was provided as an enrichment material in each pen.

There were three treatments: (i) pigs vaccinated against Porcine Circovirus type 2 (PCV2) by means of intradermal vaccination with Porcilis^®^ PCV ID (IDAL); (ii) pigs vaccinated against PCV2 with Porcilis^®^ PCV intramuscularly with a needle (IM) (iii) a control group where pigs were managed identically but did not receive any vaccine (CONTROL). There was a total of 15 pens, meaning 5 pens per treatment randomly allocated through the room but avoiding two consecutive pens from the same treatment. Vaccinations were performed by trained persons. At the time of vaccination, all pigs were grabbed from their hind legs, handled and vaccinated according to the treatment schedule (control pigs were touched with the hand).

### 2.3. Behavioural Measures

At the time of injection retreat attempts (absence = piglet remains still; presence = piglet moves during vaccination and stops moving or continues to move after the injection has ceased) and high pitch vocalizations (absence = piglet does not vocalize or piglet grunts; presence = piglet screaming or squealing) were recorded at the individual level in a subsample of 84 pigs (28 pigs per treatment, between 5–6 pigs per pen) randomly sampled within each pen. Those pigs were randomly selected and marked with a color sticker on the back area before starting the trial.

Social behaviour, general activity and resting postures were recorded at pen level and by direct observation 24 h before and after the vaccination procedure. Social behaviour and general activity were recorded by scan sampling adapted from the methodology proposed by the Welfare Quality^®^ (WQ^®^) protocol for growing pigs on the farm [26], which is briefly described here. Pigs were scored as either active or inactive. The behaviours recorded from active pigs are shown in Table 1 and followed the methodology described by Temple et al. [11]. Observations took place the day before and the day after the vaccination procedure, with three observations blocks per day for each pen (from 9 a.m. to 4 p.m.). Within an observation block, each pen was observed 5 times with a 2.5 min interval between two scans. The number of pigs engaged in each social behaviour category (social negative and social positive) and four active behaviours (feeding, drinking, exploration and “other”) were recorded. Social behaviour, feeding, drinking, exploration and “other” behaviour were expressed in proportion to the total number of active pigs. The percentage of active pigs was expressed as the proportion of the total number of observations (active + resting animals). Resting postures were recorded at the same time, by means of the same scan sampling method and counting the number of pigs lying and sitting. Moreover, when pigs were lying a determination was made as to whether they were lying ventrally or laterally (Table 1).

### 2.4. Health Measures and Body Weight

Rectal temperature and skin reaction at the point of injection were measured on the same 84 animals whose behavioural reaction at the time of injection was recorded. Rectal temperature was assessed with a thermometer on day −1, 0, +1 (+28 h), +2 (+42 h) and +7 of the vaccination. Skin reaction at the point of vaccine injection was assessed on day 0, +1 (+28 h), +2 (+42 h), +7 and +28 of the vaccination. Skin reaction was evaluated visually and by palpation. A lesion at the injection site was considered as present when observing a slight redness of more than 0.20 cm or any induration at palpation.

Piglets were weighed on day 0 (the day of vaccination treatment) and day +21 after vaccination.

### 2.5. Sampling of Physiological Parameters

The same eighty-four pigs were sampled for salivary parameters. Saliva samples were obtained through a small sponge that the piglet grabbed during at least 30 s. During a one-week adaptation period before starting the trial, piglets were trained to grab the small sponge without showing any fear reaction and to ensure getting a sufficient quantity of saliva. Conditioning of each individual piglet was achieved using sponges soaked in a solution of milk replacer and honey. Conditioning was considered sufficient when the piglet griped the small sponge (without milk and honey) without showing any fear reaction. Two saliva samples were obtained from each pig, one immediately before vaccination and a second one 25 min after vaccination. The sponges were centrifuged in a plastic tube at 3× *g* for 10 min at 4 °C. Saliva samples were then aliquoted and stored at −22 °C until analysis. Salivary CgA and sAA activity were determined through assays validated and described in earlier studies [14,15].

The same 84 pigs were blood sampled on day 0 (20–30 min after vaccination) as baseline and 28 h post-vaccination for APP determination. Blood sampling for baseline level was done 20–30 min after vaccination, once saliva sampling was over, to avoid any interference with the saliva sampling. Blood was extracted from the jugular vein, centrifuged at 2× *g* for 5 min and supernatant kept at −80 °C until analysis. Porcine C-Reactive Protein (CRP) and Haptoglobin (Hp) levels were measured by an automated biochemistry analyser (Olympus AU2700, Olympus Diagnostica GmbH, Hamburg, Germany) previously validated in pigs [28,29].

### 2.6. Statistical Analysis 

Data were analysed using the statistical package SAS (SAS.9.4. Institute Inc., Cary, NC, USA). Normality of residuals was checked through the Shapiro-Wilk test and QQ (quantile-quantile) plots of residuals for each one of the dependent variables studied. When a variable met the normality assumption, a Linear Model was used.

The pen was the statistical unit for all behavioural data and postures obtained by instantaneous scan sampling. Negative social behaviour, positive social behaviour, exploration of the pen features, exploration of the rope, drinking and eating were expressed in proportion to the total number of active pigs. The percentage of active pigs was expressed in proportion to the total number of observations (active + sleeping animals). All these behavioural variables met the normality assumption and were analysed with Linear Models. The occurrence of postures (lying, lying ventrally and sitting) did not meet the normality assumption and were analyzed by means of Generalized Linear Models applying a negative binomial distribution. The models included the main effect of the treatment (3 levels: CONTROL, IDAL, IM) and the initial level at day −1 was included as a covariate and referred to as the baseline.

The proportion of pigs showing high pitch vocalizations (absence/presence) and retreat attempts (absence/presence) at the time of vaccine injection in the IM and the IDAL groups was compared using Fishers’ Exact tests.

Physiological data (CgA, sAA, Hp, and CRP), as well as rectal temperature and Average Daily Gain (ADG), were tested applying Linear Models. sAA and CRP were log-transformed to reach normality. The models included the main effect of the treatment (3 levels: CONTROL, IDAL, IM) and the initial level was included as a covariate and referred to as the baseline.

The proportion of pigs showing a skin reaction at the site of injection (absence/presence) in the IM and the IDAL groups was compared using Fishers’ Exact tests.

A *p*-value of 0.05 was considered significant for all analyses.

## 3. Results

### 3.1. Behavioural Data

The frequency of pigs showing retreat attempts (Table 2) at the time of injection was significantly higher in the IM treatment (39%) compared to the IDAL (7%, *p*-value = 0.009, Fisher’s exact test) and the CONTROL groups (3%, *p*-value = 0.001, Fisher’s exact test). The frequency of pigs showing high pitch vocalizations (Table 2) at the time of injection was significantly higher in the IM treatment (32%) compared to the IDAL (7%, *p*-value = 0.04, Fisher’s exact test) and the CONTROL groups (3%, *p*-value = 0.02, Fisher’s exact test ). IDAL piglets and CONTROL piglets did not differ in terms of retreat attempts and high pitch vocalizations.

Table 3 shows the effect of IDAL vaccination on social and exploratory behaviour, general activity and resting postures. The day after vaccination, piglets vaccinated intramuscularly showed a significant decrease of social negative interactions compared to the IDAL (*p*-value = 0.001) and CONTROL (*p*-value = 0.001) groups. IM piglets explored the rope significantly less frequently after the vaccination compared to the CONTROL group (*p*-value = 0.04). Finally, a significant decrease in total activity was seen in the IM group compared to the IDAL (*p*-value = 0.04). The remaining active behaviours studied did not vary significantly across treatments. Resting postures (Table 4) as well as the percentage of huddling animals (IM day + 1 = 6.9 ± 3.03% vs. IDAL day + 1 = 7.6 ± 3.99% vs. CONTROL day + 1 = 4.1 ± 3.12%) were also not affected by the type of vaccination procedure.

### 3.2. Physiological Data

Pigs from the IM treatment presented significantly higher levels of blood CRP and blood Hp, 28 h after vaccination, compared to the IDAL and the CONTROL groups (Table 5). Salivary sAA and CgA levels were not affected by the type of vaccination procedure. sAA and CgA levels did not vary before and after vaccination (Table 6).

### 3.3. Health Data and Body Weight 

The prevalence of pigs with local reactions (skin alteration) altered skin alteration at the site of injection is shown in Table 7. At palpation, a skin reaction of 0.2–0.5 cm diameter was observed in 18% of IDAL piglets at +28 h while 1% of pigs vaccinated intramuscularly presented a skin reaction at the site of injection.

Rectal temperature was not significantly different between treatments (Table 8).

The Average Daily Gain (ADG) did not differ significantly (*p*-value = 0.45) between treatments (Table 9).

## 4. Discussion

The present study evaluated the effect of the needle-free intradermal IDAL vaccination method on the welfare of weaned piglets through behavioural, physiological and health parameters.

Currently, commercially vaccines against swine-relevant pathogens are licensed for the intradermal needle-free delivery route for diseases such as *Mycoplasma hyopneumoniae*, Porcine reproductive and respiratory syndrome virus (PRRSV), Porcine circovirus (PCV) 2, and Pseudorabies virus. In our study, PCV2 was chosen for its high clinical impact and huge economic losses. This pathogen is ubiquitous across pig farms [30]. Due to its importance in the swine industry and the high efficacy demonstrated by vaccines since the first one was marketed more than ten years ago, PCV2 vaccine is one of the most widely used vaccines in the largest pig producing countries [31]. Besides mechanical actions due to the presence or absence of a needle, the effect produced by the administration of large volumes and irritating substances are important factors that affect the degree of pain during administration of substances [1]. The intradermal route of administration uses much smaller volumes of substances than the intramuscular route [18]. Thereafter, if the excipient administered has similar properties across vaccines, the welfare benefits of needle-free vaccination against PCV2 should be, in part, applicable to other types of pig needle-free vaccines as well. Still, further studies would be required to ensure such extrapolation.

This study is part of a wider project where the immune response of the piglets to different vaccination methods was evaluated. All pigs were seronegative to PCV2 prior to vaccination and the control pigs did not seroconvert after vaccination. Both intramuscular and intradermal vaccination induced a clear and detectable humoral and cellular immune response, indicating that both vaccination routes induced a solid immune response [32]. Therefore, it is rather unlikely that the behavioural and physiological differences observed between vaccination methods in the present study would be linked to differences in the immune response.

The day after vaccination, pigs vaccinated intramuscularly (IM) showed a decreased activity compared to the intradermal group. Similar results were reported in a previous study in sows [11]. Göller et al. [9] found that piglets vaccinated intradermally were more active at the udder than IM vaccinated piglets, which was associated with a reduced degree of stress. Piglets suffering discomfort or pain may indeed show altered behavioural patterns [33]. The reduced levels of negative social behaviours and exploration in pigs vaccinated IM may indicate some degree of discomfort or pain the day after the IM vaccination. Intradermal vaccination did not seem to alter the behaviour of piglets one day after vaccination.

Different call types such as grunts, squeals or screams in piglets can be used as evidence for pain-related vocalizations [34]. The percentage of pigs displaying high pitch vocalizations and retreat attempts at the time of injection were higher in the piglets vaccinated IM and no difference were observed between the IDAL and control piglets. Those observations were done through direct observations. A similar fear/pain reaction at the time of the injection was observed in sows, even though this difference between vaccination methods appear to be more apparent in sows than piglets [11]. Using a digital sound level meter and audio editing software, Scollo et al. [10] reported a greater number of vocalizations emitted by piglets vaccinated IM, with a longer and more powerful peak. Altogether, these studies suggest that intradermal vaccination reduces the acute pain reaction of pigs during the injection.

Salivary alpha-amylase (sAA) and salivary chromogranin A (CgA) levels were not affected by the type of vaccination procedure. Basal sAA and CgA levels were similar to levels after vaccination in all three treatment groups, including the control group. Therefore, the handling procedure itself for vaccination did not seem to affect sAA and CgA levels. sAA levels observed in this study were similar to the low sAA activity levels (without stressor) [16]. On the contrary, CgA levels before and 30 min after the vaccination procedure were comparable to the levels of CgA of piglets after isolation (1.5–1.6 µg/mL) [15]. Previously to the salivary sampling, during a one-week training, all piglets were habituated and conditioned individually to grab the small sponge. This training was carried out to avoid any fearful reaction from the piglets to the saliva sampling and to maximize the volume of saliva collected. The day of vaccination, the piglets were so conditioned to grab the small sponge that they were very excited just seeing the sponge. Therefore, the sampling procedure through positive training induced a high level of arousal in the piglets that may have biased the levels of salivary stress biomarkers of SAM activity.

The acute phase proteins (APPs), are a group of blood proteins that change in concentration in animals subjected to external or internal challenges, such as infection, inflammation, surgical trauma and stress [35]. CRP has been used to assess acute inflammation one day after vaccine administration [36]. As well, Hernández-Caravaca et al. [37] used CRP and Hp to evaluate the inflammatory response of pigs to vaccination. In the present study, pigs from the IM treatment presented significantly higher levels of CRP and Hp 28 h after vaccination, compared with the IDAL and the CONTROL groups. Levels of CRP and Hp in piglets vaccinated IM with the needle-syringe method were 2.1 and 1.6 times the value observed in IDAL piglets. Therefore, the inflammation caused by intramuscular needle-syringe compared to IDAL vaccination resulted in higher levels of CRP and Hp 28 h after vaccination. Needle-free injections have been reported to reduce muscular damage and carcass defects [24,25]. Lower levels of APPs in the IDAL piglets 28 h after vaccination may indicate a reduced inflammation and muscular trauma due to the injection. 

Piglets vaccinated with the IDAL method in the neck presented a higher prevalence of skin reaction at the site of injection than pigs vaccinated IM with a needle. This reaction was described as a spot of 0.2–0.5 cm diameter detected through palpation at the site of injection. In a recent study, minor local reactions were also observed in sows after intradermal vaccination [38]. Contrary to IDAL vaccination, the deposition of the vaccine in the IM group is into the muscle and any local reaction may not be readily visible without undergoing necropsies. Days later, no abscess-like reaction was observed in any of the two vaccinated groups. In sows, more than 20% of the animals vaccinated intramuscularly presented abscess-like reactions 28 days post-vaccination [11]. Rectal temperature was not significantly different between treatments. This is in accordance with other studies where the intradermal application (in that case of Porcilis^®^ PRRS) did not induce systemic reactions or any increase in rectal temperature [38]. The method of vaccination and the vaccination by itself did not alter the ADG.

## 5. Conclusions

In this study, the needle-free intradermal vaccination against PCV2 reduces piglets’ retreat attempts and high pitch vocalizations at the time of injection compared to the intramuscular vaccination. Contrary to intramuscular vaccination, intradermal vaccination did not affect general activity, social behaviour and exploratory behaviour of the piglets after the injection. At 28 h post-vaccination, intradermal piglets presented lower blood C-reactive protein and blood Haptoglobin levels compared to piglets vaccinated intramuscularly. Needle-free intradermal vaccination may therefore prevent the acute phase response and muscular damage associated with intramuscular injections. The method of vaccination did not affect the growth of the piglets and their rectal temperature. The needle-free intradermal method of vaccination of pigs represents a less invasive and less painful alternative to conventional intramuscular injections with needles. The welfare benefits of needle-free vaccination of piglets without catching the animals should be further investigated.

## Figures and Tables

**Table 1:** Description of behavioural variables and postures assessed during direct observations (scan sampling) at 24 h pre- and post-vaccination.

Behavioural Category	Definition
Active behaviour	
Negative social behaviour	Aggressive behaviour, including biting or any social behaviour with a response from the disturbed animal (from the Welfare Quality WQ protocol for growing pigs [26])
Positive social behaviour	Sniffing, nosing, licking and moving gently away from the animal without an aggressive or flight reaction from this individual (from the WQ protocol for growing pigs [26])
Drinking	Mouth on water trough (from Mc Guy et al. [27])
Eating	Mouth on feeder (from Mc Guy et al. [27])
Exploring pen features	Sniffing, nosing, licking or chewing all features of the pen (from the WQ protocol for growing pigs [26])
Exploring enrichment material (organic rope)	Sniffing, nosing, licking or chewing an organic rope (from the WQ protocol for growing pigs [26])
Other active behaviours	All other active behaviours (air sniffing, gazing, walking, etc.)
Sleeping	Sleeping (lying down with the eyes shut)
Posture	Definition
Standing	Bodyweight supported by all four legs
Sitting	Bodyweight supported by hindquarters and front legs
Lying ventrally	Bodyweight supported by belly and sternum in contact with the floor
Lying laterally	Bodyweight supported by side with the shoulder in contact with the floor

**Table 2:** Percentage of pigs showing retreat attempts and high pitch vocalizations at the time of injection in the CONTROL, Intradermal (IDAL), and Intramuscular (IM) groups.

Variable	CONTROL	IDAL	IM
% of pigs with high pitch vocalization	7% ^b^	7% ^b^	32% ^a^
% of pigs with retreat attempts	3% ^b^	7% ^b^	39% ^a^

^a,b^ Different letters indicate significant differences between treatments (CONTROL; IDAL; IM).

**Table 3:** Mean percentages of occurrence and Standard Deviation (SD) of behaviours recorded in the CONTROL, IDAL, and IM groups.

Social Behaviour and General Activity (% of Events)	CONTROL	IDAL	IM
Social negative			
Before (Day − 1, baseline)	11.4 ± 12.45	9.5 ± 3.29	10.1 ± 4.25
After (Day + 1)	8.1 ± 3.18 ^a^	8.0 ± 3.22 ^a^	3.4 ± 3.48 ^b^
Social positive			
Before (Day − 1, baseline)	12.1 ± 2.44	14.3 ± 6.50	12.6 ± 2.81
After (Day + 1)	12.8 ± 3.46	14.7 ± 5.89	11.0 ± 8.12
Drinking			
Before (Day − 1, baseline)	2.5 ± 1.51	3.9 ± 2.70	2.8 ± 2.50
After (Day + 1)	2.5 ± 2.19	2.8 ± 3.31	3.5 ± 3.10
Eating			
Before (Day − 1, baseline)	20.3 ± 4.79	23.2 ± 8.31	22.6 ± 6.76
After (Day + 1)	26.6 ± 6.08	26.0 ± 7.20	28.2 ± 11.27
Exploration pen			
Before (Day − 1)	20.9 ± 6.31	17.6 ± 6.58	19.4 ± 4.84
After (Day + 1)	19.9 ± 5.8	18.3 ± 4.92	14.65 ± 11.32
Exploration rope			
Before (Day − 1, baseline)	5.6 ± 4.07	5.8 ± 6.27	3.8 ± 2.21
After (Day + 1)	5.7 ± 4.07 ^a^	4.8 ± 3.16 ^ab^	2.2 ± 2.77 ^b^
Active total			
Before (Day − 1, baseline)	59.5 ± 10.72	46.3 ± 13.50	54.3 ± 12.18
After (Day + 1)	47.6 ± 17.77 ^ab^	47.8 ± 15.72 ^a^	34.9 ± 23.35 ^b^

^a,b^ Different letters indicate significant differences between treatments (CONTROL; IDAL; IM).

**Table 4:** Resting postures expressed as a median and interquartile range of occurrence in the CONTROL, IDAL and IM groups.

Resting Pattern (% of Events)	CONTROL	IDAL	IM
Lying total			
Before (Day − 1, baseline)	55 (26)	75 (18)	66 (24)
After (Day + 1)	67 (27)	75 (25)	84 (24)
Lying ventrally			
Before (Day − 1, baseline)	49 (15)	61 (14)	56 (17)
After (Day + 1)	58 (21)	64 (17)	66 (27)
Sitting			
Before (Day − 1, baseline)	2 (2)	1 (2)	1 (1)
After (Day + 1)	0 (1)	1 (1)	0 (1)

**Table 5:** Means, SD and coefficient of variation (CV) of blood acute phase proteins CRP and Hp for the CONTROL, IDAL and IM treatments on day 0 (baseline) and day +1 (28 h) after the vaccination.

Acute Phase Protein		CONTROL	IDAL	IM
CRP (µg/mL)	t0	12.9 ± 9.12 (70%)	9.7 ± 12.60 (120%)	11.1 ± 8.97 (80%)
	t1 (+28 h)	20.0 ± 15.12 ^c^ (75%)	38.8 ± 33.13 ^b^ (87%)	82.8 ± 29.18 ^a^ (35%)
Hp (mg/mL)	t0	1.8 ± 0.62 (34%)	1.7 ± 0.72 (43%)	1.8 ± 0.72 (41%)
	t1 (+28 h)	1.8 ±0.65 ^b^ (35%)	1.9 ± 0.71 ^b^ (37%)	3.1 ± 0.3 ^a^ (9%)

^a–c^ Different letters indicate significant differences between treatments (CONTROL; IDAL; IM).

**Table 6:** Means, SD and coefficient of variation (CV) of salivary alpha-amylase (sAA) and salivary Chromogranin-A (CgA) for the CONTROL, IDAL, and IM treatments just before vaccine injection (t0, baseline) and 25 min after vaccine injection (+25 min).

Salivary Parameter		CONTROL	IDAL	IM
Alpha-amylase (sAA) (UI/L)	t0	793.3 ± 1288.68 (162%)	656.8 ± 867.34 (132%)	549.8 ± 714.76 (130%)
	t1 (+25 min)	462.3 ± 970.36 (210%)	644.8 ± 1002.40 (156%)	785.6 ± 1456.4 (185%)
Chromogranin-A (CgA) (μg/mL)	t0	1.19 ± 0.36 (30%)	1.26 ± 0.41 (32%)	1.34 ± 0.30 (22%)
	t1 (+25 min)	1.35 ± 0.40 (29%)	1.43 ± 0.32 (22%)	1.36 ± 0.36 (26%)

**Table 7:** Mean percentage of pigs from the CONTROL, IDAL, and IM groups showing a local reaction at the site of the injection +28 h, +42 h and 21 days post-vaccination.

Group	+28 h	+42 h	+7 days	+21 days
CONTROL	0	0	0	0
IDAL	18%	16%	11%	7%
IM	1%	1%	1%	1%
*p*-value between IDAL and IM	<0.0001	0.0001	0.012	0.10

**Table 8:** Mean rectal temperature of pigs from the CONTROL, IDAL and IM groups on day − 1, +28 h, +42 h, 7 days, 21 days post-vaccination.

Group	Day − 1 (Baseline)	+28 h	+42 h	+7 days	+21 days
CONTROL	39.3 ± 0.52 °C	39.4 ± 0.47 °C	39.3 ± 0.35 °C	39.4 ± 0.48 °C	39.9 ± 0.35 °C
IDAL	39.3 ± 0.39 °C	39.4 ± 0.44 °C	39.1 ± 0.49 °C	39.1 ± 0.72 °C	39.8 ± 0.53 °C
IM	39.5 ± 0.53 °C	39.5 ± 0.41 °C	39.2 ± 0.44 °C	39.3 ± 0.59 °C	39.7 ± 0.32 °C

**Table 9:** Mean body weight the day of the vaccination (Day 0) and 21 days after the vaccination.

Group	BW day 0 (kg)	BW day + 21 (kg)	ADG (kg)
CONTROL	10.6	20.3	0.44
IDAL	9.6	18.3	0.42
IM	9.2	18.4	0.44
